# A Rare Case of Isolated Hepatocellular Carcinoma Metastasis in Left Mandibular Region in a Patient with Hepatitis C Virus Liver Cirrhosis Diagnosed after the Onset of COVID-19 Infection

**DOI:** 10.3390/medicina59111992

**Published:** 2023-11-13

**Authors:** Dragan Mašulović, Aleksa Igić, Aleksandar Filipović, Miloš Zakošek, Dušan Bulatović, Ksenija Mijović, Marjan Micev, Danijel Galun

**Affiliations:** 1 Center for Radiology, University Clinical Center of Serbia, Pasterova No. 2, 11000 Belgrade, Serbia; draganmasulovic@yahoo.com (D.M.); aleksandar.filipovic11@gmail.com (A.F.); zaki0502@gmail.com (M.Z.); dusan.bulatovic1@gmail.com (D.B.); mijovicksenija@gmail.com (K.M.); 2 Department of Radiology, Faculty of Medicine, University of Belgrade, Dr Subotica No. 8, 11000 Belgrade, Serbia; galun95@gmail.com; 3 Department for Pathology, Clinic for Digestive Surgery, University Clinical Centre of Serbia, Dr Subotica No. 8, 11000 Belgrade, Serbia; micevm@gmail.com; 4 HPB Unit, Clinic for Digestive Surgery, University Clinical Center of Serbia, 11000 Belgrade, Serbia

**Keywords:** hepatocellular carcinoma, mandible, metastasis, biopsy, diagnosis

## Abstract

*Background and Objectives:* Hepatocellular carcinoma (HCC) most frequently metastasizes in the lungs, abdominal lymph nodes and adrenal glands. Metastatic spread to the head and neck area is extremely rare. In the presented case, an uncommon site of solitary metastatic spread of HCC to the mandible confirmed after the core biopsy of the lesion is reported. There have been only about 80 cases of mandibular HCC metastases described in the literature to date. We contribute our experience to the pool of data. *Case presentation:* A 65-year-old female with HCV-related liver cirrhosis was diagnosed with an HCC that was successfully treated with liver resection. Subsequently, the patient had developed COVID-19 disease, which was associated with a painless swelling in the left jaw. A neck MDCT scan demonstrated an osteolytic soft-tissue mass in the left mandible, with the characteristics consistent for the metastasis of HCC. In order to confirm the diagnosis, a core biopsy of the mandibular mass was performed. The pathohistological evaluation confirmed the presence of a metastatic HCC in the mandible. No other sites of disease dissemination were identified in extensive MDCT scans. Despite considering various treatments, including symptomatic and palliative, the patient’s overall prognosis remained poor. *Conclusions:* Isolated metastases of HCC to the orofacial region are extremely rare; however, it should be considered in patients with known risk factors for HCC development. Early diagnosis is critical, and clinicians should consider this possibility of HCC spread when assessing patients with orofacial swelling, among those patients with risk factors for HCC. The overall prognosis for such patients remains poor, emphasizing the challenges in managing these cases.

## 1. Introduction

The liver is the sixth most common site of primary malignancies, with nearly 1 million new cases diagnosed each year (4.7% of all sites); liver cancer is the third most common cause of cancer-related deaths following lung and colorectal cancers [1]. Hepatocellular carcinoma (HCC) is the most common primary liver cancer, comprising more than 70% of primary liver malignancies, with risk factors widely dependent on geographic and ethnic variations. The most common risk factors are viral hepatitis (HBV, HCV), as well as other known risk factors including aflatoxin, alcohol abuse and smoking, or metabolic disorders such as non-alcoholic fatty liver disease (NAFLD), obesity and type 2 diabetes [1,2].

The most common sites of HCC metastasis are the lungs, abdominal lymph nodes, adrenal glands and bones, while the brain, peritoneum, skin and muscle are rarely affected. The increasing prevalence of extrahepatic metastases can be attributed to advancements in diagnostic techniques and the extended survival of patients, owing to the evolution of various treatment options available for primary HCC [3]. Maxillofacial metastases are quite rare, and when they do occur, they primarily affect the mandible and less frequently the maxilla and other regions [4]. The largest literature review so far, dating from 2007, summarized only 17 reports of metastatic HCC in the sinonasal region, and another 86 reports of it in the various other regions of the head and neck area, making up a total of 103 HCC metastases in the head and neck region including the sinonasal area [5]. Moreover, solitary orofacial HCC metastases are extremely rare, and they are considered to occur via an alternative hematogenous route and may present the initial symptom of the disease, which is associated with poor prognosis.

We present a rare case of a solitary extrahepatic metastasis of a HCC in the mandible, confirmed after a core biopsy of the lesion.

## 2. Case Report

**Figure 1:**
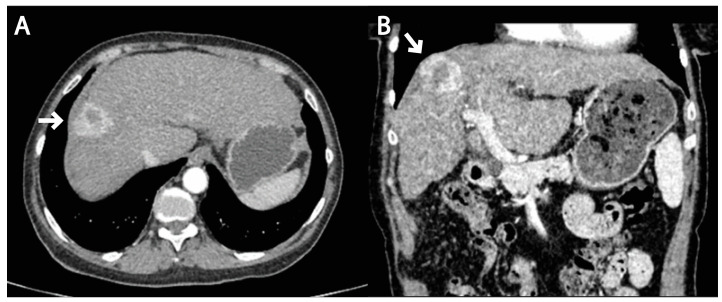
Axial (**A**) and sagittal (**B**) images of a contrast-enhanced CT scan obtained in the arterial phase reveal the presence of a solitary, hypervascular liver tumor in segment VIII of the liver (arrows), with washout in portal venous phase (not shown in these images), consistent with the radiological features of an HCC in the setting of a cirrhotic liver.

A 65-year-old female patient was admitted to the surgical clinic with previous computed tomography (CT)-detected presentation of a subcapsular 3 cm solitary, hypervascular, primary liver tumor in segment VIII, in the setting of a cirrhotic liver caused by chronic HCV infection, highly suggestive of an HCC (Figure 1). The CT scan demonstrated signs of portal hypertension, such as recanalization of paraumbilical veins and discrete venous collaterals in the omentum and esophageal varices. Additionally, there was no evidence of extrahepatic disease in the thorax, abdomen or pelvis, nor signs of ascites. Upon admission to the hospital and detailed clinical and laboratory work-up, the patient was classified as Child–Pugh class A, with the following values: total bilirubin level of 20.2 mmol/L, albumin level of 39 g/L, International Normalized Ratio (INR) of 1.16, without signs of ascites or encephalopathy. Furthermore, the patient’s performance status, assessed via the Eastern Cooperative Oncology Group (ECOG) grading system [6], was 0. Considering the patient’s favorable performance status, without comorbidities, and Child–Pugh class A, the patient met the stage A criteria of the Barcelona Clinic Liver Cancer (BCLC) system [7], and curative-intent surgical intervention (open anatomic resection of liver segment VIII) was performed. During the surgical procedure, a subcapsular tumor of approximately 3 cm in size, localized in segment VIII of the liver, was observed, which was protruding on the surface of the liver parenchyma and adhering to the peritoneal lining of the right hemidiaphragm. This finding was confirmed with an intraoperative ultrasound. Initially, a part of the hemidiaphragm around the tumor was resected without opening the right hemithorax, and then further resection of segment VIII of the liver was performed using radiofrequency resection (RFS), along with the ablation of the pedicle for the mentioned liver segment. The resected area showed no signs of bleeding and was hemostatically controlled. The pathohistological (PH) report of the resected specimen confirmed the presence of a moderately differentiated G2 trabecular-type HCC, with a pathological Tumor–Node–Metastasis (pTNM) staging in the IB prognostic group, which consists of T1b (solitary tumor > 2 cm without vascular invasion), N0 (no regional lymph node metastasis) and M0 (no distant metastases). The surgical margin was microscopically negative for residual tumor—R0 resection (no residues of the tumor on the surgical margin). The postoperative course was uneventful, and the patient was discharged from hospital on postoperative day 7. The first follow-up visit was scheduled for one month and furthermore on a 6-month basis. Given the curative R0 resection, no chemo- or radiotherapy were advised.

**Figure 2:**
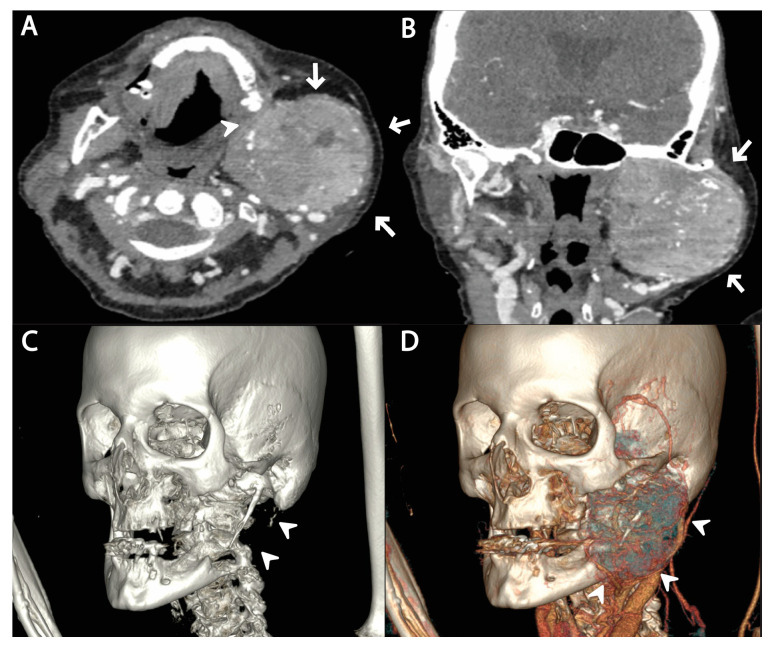
Axial (**A**) and sagittal (**B**) images of contrast-enhanced neck CT scan show a large, lobulated and intensively opacified osteolytic soft-tissue mass in the left mandible angle and ramus (arrows), extending from the base of the skull to the mandibular angle. Infiltration of masticatory muscles is observed as well. The volume-rendered images show destructed left mandibular ramus and mandible angle (arrowheads) due to tumor invasion (**C**), as well as an image of vascular 3D reconstruction, which shows highly vascularized tumor (arrowhead) (**D**).

Two months after the initial diagnosis and subsequent liver resection, the patient was admitted to a COVID-19 hospital due to a positive PCR COVID-19 test and chest X-ray findings of bilateral pneumonia. At the admission, she reported a diffuse, firm and painless swelling in the left jaw, which she noticed a week prior to admission, and thereafter a neck CT was ordered. The neck CT demonstrated a large osteolytic soft-tissue mass in the left mandibular angle and ramus, spreading to the temporal fossa and parotid area occupying the space from the skull base to the submandibular area. The mass displayed heterogeneous and intense contrast enhancement, along with osteolytic bone changes and infiltration of the masticatory muscles (Figure 2).

**Figure 3:**
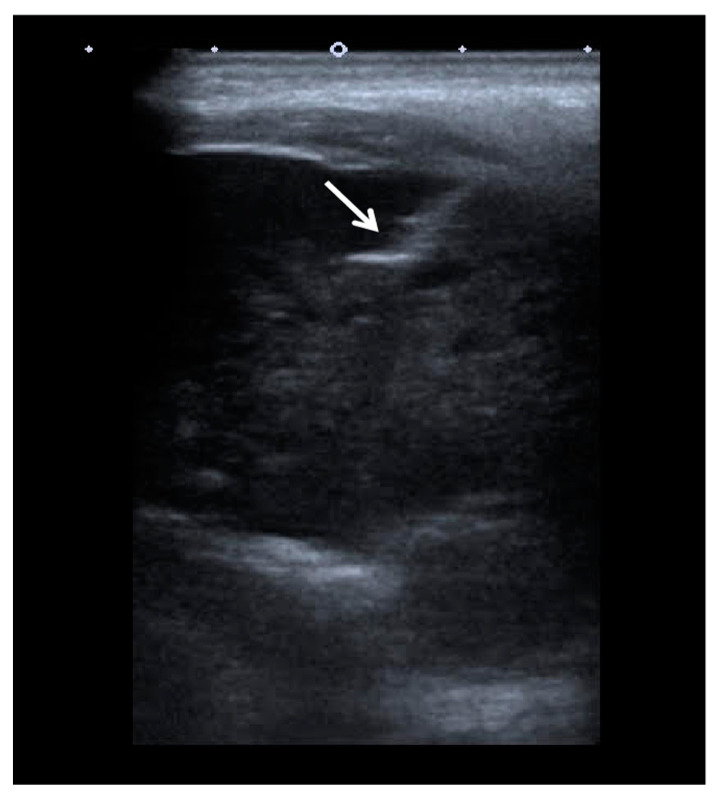
The ultrasound image of the left mandibular region mass obtained during the percutaneous ultrasound-guided core needle biopsy (arrow) shows the core biopsy needle inside the left mandibular region mass.

**Figure 4:**
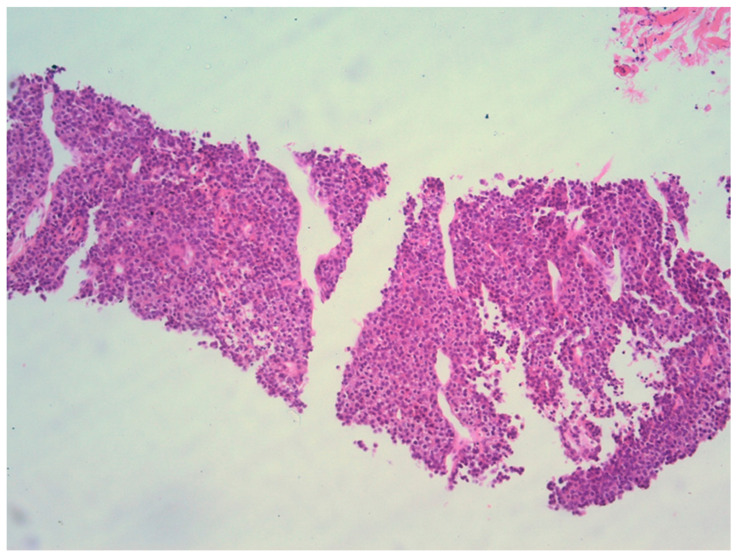
Percutaneous core needle biopsy sample of the tumorous infiltration in mandibular region reveals histological findings completely consistent with previously diagnosed trabecular type of HCC.

The patient was referred to the Department of Interventional Radiology in consideration of a percutaneous biopsy of the mass. The core biopsy of the mass in the left mandibular region was performed under ultrasound guidance (Figure 3) using an 18-gauge disposable guillotine spring-loaded needle for biopsy of soft tissue (BD, Biomedical Srl, Firenze, Italy). Pathohistological (PH) findings revealed a metastatic carcinoma in the left mandibular region, completely consistent with the previously diagnosed trabecular type of hepatocellular carcinoma (Figure 4). There were no other sites of disease dissemination upon an endocranial, neck, chest, abdominal and pelvic CT performed shortly after. The maxillofacial oncological team concluded that due to the prolonged recovery from COVID-19 disease, as well as due to the accelerated disease progression and poor prognosis of patients with extrahepatic disease, the surgical treatment of the mandible lesion was not indicated, and the treatment was continued with symptomatic and palliative treatment. Ten months later, the patient died due to hepatic decompensation and local spread of the metastatic disease. However, there were no clinical or radiological signs of recurrent disease in the liver.

## 3. Discussion

Advances in imaging techniques and in the treatment of HCC patients with subsequent prolonged survival allow us to observe extrahepatic metastases more frequently and earlier than we could before [3]. The incidence of extrahepatic metastases is estimated to be 13–36% [8], with the most frequent sites being the lungs (18–55%); lymph nodes (26.7–53%); bones, most commonly including the axial skeleton, (5.8–38%); and the adrenals (8.4–15.4%) [3], while mandibular metastasis is estimated to occur in 0.6% of cases [9]. Extrahepatic metastases of HCC tend to affect males, particularly in patients aged over 60 years, exhibiting the traits of the primary tumor [4].

Metastases to the head and neck region are exceptionally rare, and the initial report of metastatic hepatocellular carcinoma in the mandible was described by Dick A. et al. back in 1957 [10]. Since then, there have been approximately 80 documented cases of metastasis to the mandible with the most extensive literature review to date summarizing 77 of these cases [11]. HCC metastasizes hematogenously through tumor cell invasion of hepatic artery or portal venous branches, with subsequent seeding of the lungs as the most common site of metastatic disease. Therefore, oral metastases are mostly associated with lung metastases, probably occurring via the same hematogenous route [12]. Nevertheless, isolated metastases in the orofacial region, as occurred in the presented patient, require another explanation of the spread of the disease. In these cases, Batson’s valveless venous vertebral plexus was suggested as an alternative route of hematogenous disease spread. This plexus with various anastomoses allows the tumor cells to bypass the caval veins and reach the vertebrae and even the bones of the head and neck before reaching the right heart and the lungs. This route of metastatic disease was first suggested by Batson in 1940, who explained this vertebral venous plexus as the route for the “aberrant” or “paradoxical” metastases of prostate or breast cancers by performing a series of experiments on cadavers [13]. This is the most probable explanation for vertebral metastases, which are, as opposed to orofacial ones, quite common.

Commonly reported clinical findings in patients with a mandibular metastasis of HCC are those of a diffuse, firm and painless swelling in the temporal region [14], which are consistent with our patient. Additionally, symptoms including difficulty in opening of the mouth, numbness of the surrounding regions as well as toothache are observed [4,9,10,15,16]. Since there have been various reports of initial presentation of a metastatic HCC with a solitary orofacial metastasis [15,16], even more commonly than after an HCC diagnosis is established [9,16,17], we emphasize the need to consider this possibility in patients with known risk factors for HCC (liver cirrhosis, viral hepatitis and others). However, metastatic disease represents ~1% of all malignancies of the oral area [4].

Typically, the radiologic presentation of HCC bone metastasis is an osteolytic soft-tissue mass, commonly hypervascular like the primary HCC itself [18]. In this respect, it is understandable why the lesion itself is prone to bleeding [17,19]. Due to the aforementioned bone metastases with HCC characteristics, special care needs to be taken while performing biopsy; therefore, percutaneous core biopsy or fine needle aspiration (FNA) biopsies are favorable compared to incisional biopsies [9]. This characteristic imaging presentation of bone HCC metastasis was consistent in the presented patient, as evidenced through CT performed in the investigation of a painless jaw swelling. The neck CT demonstrated a large, infiltrative, hypervascular, osteolytic lesion in the left mandible ramus. Subsequently, percutaneous core biopsy of the left jaw mass, as well as a PH report, proved it to be an HCC metastasis in the mandibular bone.

Considering the general conditions of these patients, their liver disease and the proneness to bleeding of these mandibular lesions as well as their infiltrative nature, it is challenging to undertake radical procedures and complete resection; therefore, the probability of complete removal of both the primary and metastatic lesions is low [9,20]. In such cases, the only reasonable approach is a palliative treatment such as chemotherapy, radiation therapy, immunotherapy or different combinations [9,20]. Radiotherapy was considered in the presented patient; however, due to the size of the lesion, it was not indicated.

HCC typically has a grim overall prognosis, characterized by a 5-year survival rate of less than 20%, while patients with extrahepatic spread have extremely poor prognosis. [21]. Bone metastases arising from HCC lead to an extremely grim prognosis, with a median survival of just one to two months [22]. The prognosis for patients with metastatic HCC in the orofacial region is very poor, with mean overall survival of 21 weeks and a one-year survival rate of just 15% after diagnosis. Prognosis remains poor even after the application of therapy to the primary liver tumor, including surgical treatment, radiation therapy, transarterial chemoembolization (TACE) or systemic therapy with sorafenib [11,22,23].

A limitation of our case regarding the biopsy sample was that the sample contained parts of the vital part of the tumor, among other tissues, and the initial serial sectioning exhausted the paraffin tissue block. Therefore, it was not possible to perform additional immunohistochemical examinations such as immunohistochemical staining (Glypican-3 and Hep Par-1). The diagnosis was made on the basis of morphology and a comparison with the sample of previously diagnosed trabecular hepatocellular carcinoma of the liver.

## 4. Conclusions

Despite the fact that HCC rarely metastasizes to the orofacial region, these metastases are more commonly presented as the initial symptoms of the disease, before even the primary tumor has been diagnosed. This possibility should be considered in any patient with a recent swelling in the orofacial and sinonasal area, especially when there is a history of liver disease or other well-known risk factors for HCC development. Biopsy is indispensable in these cases and should not be delayed based solely on clinical grounds. The prognosis for patients with metastatic hepatocellular carcinoma in the orofacial region is notably grim, even when various treatments are applied to the primary liver tumor.

## Data Availability

All the data are available from the corresponding author upon reasonable request.
